# Editorial: Occupational immunology: current knowledge and future perspectives

**DOI:** 10.3389/fmed.2023.1322157

**Published:** 2023-11-10

**Authors:** Galateja Jordakieva, Dennis Nowak

**Affiliations:** ^1^Department of Physical Medicine, Rehabilitation and Occupational Medicine, Medical University of Vienna, Vienna, Austria; ^2^Institute and Clinic for Occupational, Social and Environmental Medicine, University Hospital, LMU Munich, Munich, Germany; ^3^Comprehensive Pneumology Center Munich (CPC-M), German Center for Lung Research (DZL), Munich, Germany

**Keywords:** occupational and environmental exposure, immune system, occupational activity, night shift work, occupational hazard factors

Immunology is a key aspect of modern occupational medicine, a field dedicated to optimizing work-related health and productivity by means of health maintenance and prevention of injury and disease. In contrast to workplace related injury, identification of health impairment associated with or resulting from occupational activity, i.e., occupational disease, is more complex. Occupational diseases can affect virtually every organ system, their development is subject to individual susceptibility and manifestation, and they are often multi-factorial, resulting from interactions between various work-related environmental and lifestyle aspects on a given genetic background. The majority of established occupational diseases are mediated by innate and adaptive immune responses, directly or indirectly linking work-related strains to health outcomes, coining the term “occupational immunology” (1).

This Research Topic aimed to highlight recent insights on occupational and work-related hazards and describe their impact on and their interrelation with the human immune system, identifying novel:

I. Work-related strains and stressors,

II. Individual susceptibility factors and diagnostic approaches, and

III. Practical aspects and considerations for occupational health and prevention.

Scientific knowledge on how emerging and established occupational hazards, e.g., allergens, chemical, physical and infectious workplace agents, impact immunological processes must be continuously expanded and updated. A recent study by Kespohl et al., revealed how current environmental stressors, i.e., climate change, promotes *Cryptostroma corticale* infestation of maple trees, a fungal infection known to trigger hypersensitivity pneumonitis (HP) in woodworkers. This work was conducted in a unique interdisciplinary collaboration between scientific institutions for prevention and occupational medicine and plant/forest protection facilities in Germany. Their research offers evaluation of diagnostic tools for serological testing and practical recommendation for preventive measures in suspected cases of *C. corticale* induced HP. In the context of diagnostic quality, Zhao et al. added the importance of comprehensively evaluating laboratory management practices based on combining established evaluation tools.

In regards to current aspirations for a precision medicine, modern occupational medicine must identify and consider factors influencing individual manifestation and susceptibility to immune mediated occupational diseases. These insights are conductive to deriving of early diagnostic procedures and updating preventive occupational health and safety measures. A meta-analysis by Word et al. addressed individual susceptibility in relation to diisocyanates, a group of versatile organic chemical compounds applied in the production polyurethane products, known to cause severe occupational asthma in a subset of exposed workers. Their research assesses connections between protein and biomarker-associated pathways before asthma development, indicating overlaps in toxicokinetic and toxicodynamic pathways, adding explicit suggestions for hypothesis-driven targeted future research in diisocyanate toxicology (Word et al.).

Beside the classical focus on immune-compromising work-related agents, modern occupational immunology must encompass other arising aspects to occupational health, such as immunological effects of shift work. Night shift work has been previously associated with conditions linked to pro-inflammatory responses, such as cardiovascular disease (2) and cancer (3). The recent HORMONIT study conducted by Harding et al. assessed immune markers, i.e., cytokines, chemokines and growth factors in plasma of male shift workers at several time points. The authors describe pro-inflammatory indicators for multiple immune response pathways based on the circadian disruption associated with night shifts, concluding an increased risk for immune dysregulation and potentially susceptibility for infections in night shift workers (Harding et al.). In line with these findings, Mohd Fuad et al. report how circadian disruption affects immunity and susceptibility to occupational toxicants exposure in night shift workers during the SARS-CoV-2 pandemic. In their review, they discuss the disruption of multiple factors affecting immunity, including the hypothalamic-pituitary-adrenal (HPA) axis, by shift work and how this affects the immunomodulatory aspects of exposure to occupational toxins and infectious agents, but also by individual health behavior, at the work place (Mohd Fuad et al.).

Other occupational aspects linked to a pro-inflammatory profile, include work-related physical inactivity levels, such as sedentary behavior (4); in contrast, introduction of moderate physical activity was shown to lead to a reduction in sickness absence in common cold season (5). While on the one hand, being physically active at work appears to promote health, high levels of occupational activity, e.g., including heavy lifting tasks, were recently associated with inflammation and mortality. In light of this emerging “physical activity paradox,” Jordakieva et al. aimed to summarize the current scientific knowledge on the link between inflammation and occupational physical activity and provide recommendations on occupational health promotion.

In summary, the scope of occupational immunology has to be regularly extended to include arising immunological stressors, including updates on immunomodulating workplace agents and encompassing any immunologically relevant factors emerging in a rapidly evolving working environment (Figure 1). Interdisciplinary insights from scientific fields complementary to occupational medicine are essential to counteracting the incidence of established and novel occupational diseases. In an era of precision medicine, monitoring and incorporating the potential impact of different occupational factors on the human immune system could add to a tailored approach to a working individual's occupational disease risk and enhance (personalized) prevention measures.

**Figure 1:**
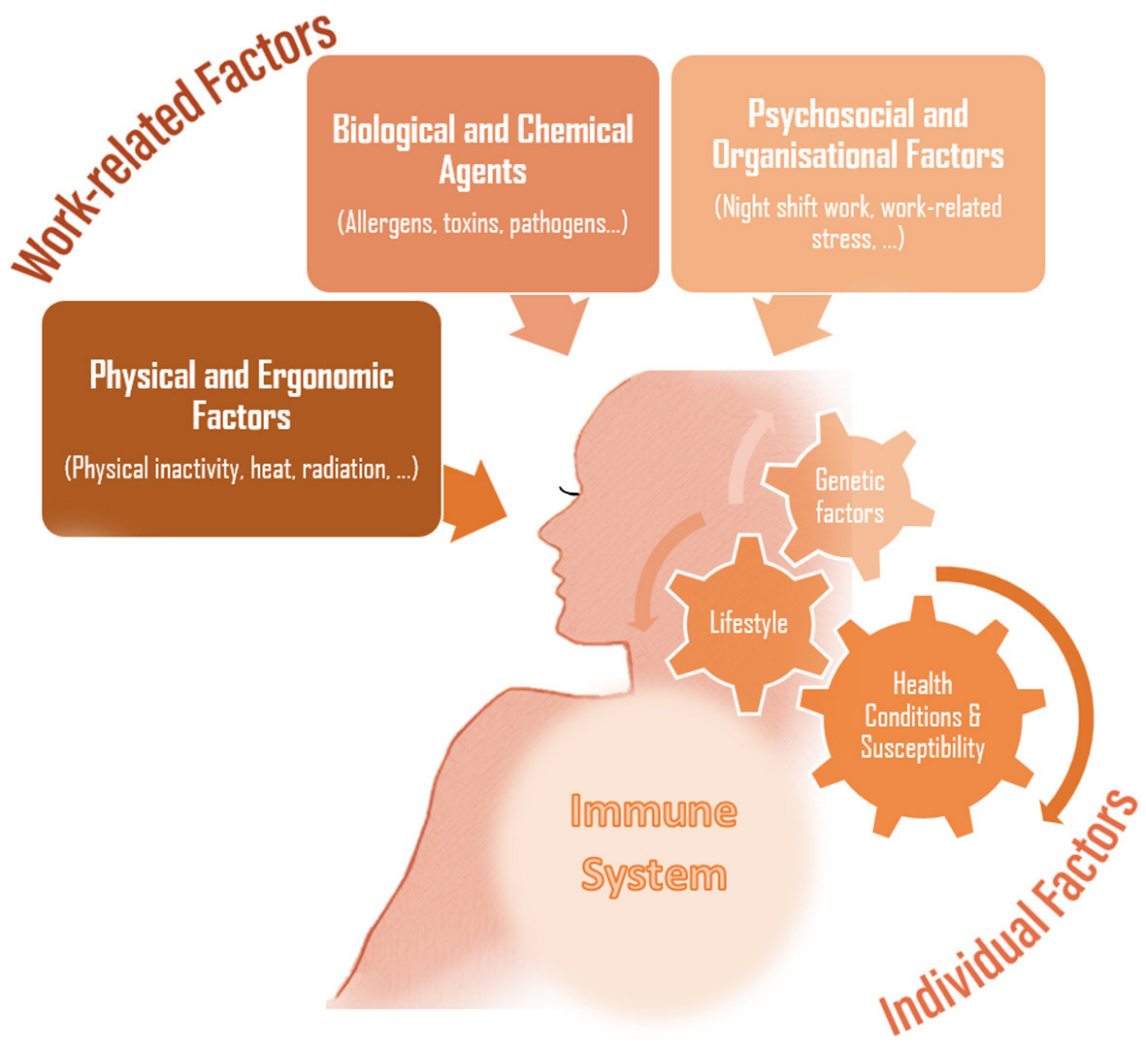
Work-related strains and stressors impacting the immune system.

## Author contributions

GJ: Writing—original draft. DN: Writing—review & editing.
